# Hypersensitivity response to the medicinal product "Zorex": clinical experience

**DOI:** 10.1186/2045-7022-4-S3-P9

**Published:** 2014-07-18

**Authors:** Marina Voronova, Olga Drobik, Elena Bashvinova

**Affiliations:** 1 Russian Medical Academy of Postgraduate Education, Russia; 2 Russian Medical Academy of Postgraduate Education, Allergy Department, Russia

## 

The medicinal product "Zorex" is currently nationally authorized in the Russian Federation. It contains unitiol and calcium pantothenate as active substances. According to the instruction for use it should be administered in a dosage of one capsule whereupon alcohol drinking to prevent the hangover or 1-2 times a day to treat the withdrawal syndrome. For the year 2013, 18 patients presented in our clinic with allergic reactions for the drug. Patients: 12 women and 6 men, 21 to 37 years old (mean age ± 28). In 16 patients such an event took place not for the first time: in 9 patients the reaction has developed for the second time, in 5 patients for the third, and in one woman for the fourth time. Clinical manifestations: in all patients symptoms have been developed after the second or third administration of the drug. All patients noted the febrile chill and the fever coming, the asthenic syndrome and in 16% of cases the large joints arthralgia at the first day whereupon the drug administration. At the end of the day, an itching skin rash appeared on the back or (less often) on the palmar surface of hand, as well as shoulders and armpit areas, buttocks and hip's internal surfaces, paraorbital or peroral areas being its the most common sites. The elements of eruption were rose-to-red spots. 88% of patients had lesions of mouth and lips mucous: small bubbles with serous contents getting opened exposing an erosion surface, painful; in some patients aphtae in the mouth cavity emerged interfering with eating and causing marked discomfort to patients. 33% of patients had vesicles on eyelids but with no lesions in conjunctive. Laboratory tests revealed leukocytosis (± 15 * 10^9^/L), and increased C reactive protein (± 20 mg/L) levels in all patients. Distinction for the condition is the regression of primary elements: at the second or the third day initial spots went to purple-bluish tint, and then the area remained persistently hyperpigmented. The hyperpigmentation persists one to two weeks after the first event. After the following events period of life of the focus is prolonged, and they persist for one-to-three months. One patient after the 4th event had a stable low-grade pigmentation. With each subsequent administration of the drug rash elements arose in place of previous eruptions, as well as a number of new ones appeared. Overall severity of symptoms also increases with repeating drug administration. All treated patients had similar clinical symptoms specific to the hypersensitivity reaction to the medicinal drug "Zorex".

